# 2-(4-Fluoro­phen­yl)-5-iodo-3-methyl­sulfinyl-1-benzofuran

**DOI:** 10.1107/S1600536809051642

**Published:** 2009-12-04

**Authors:** Hong Dae Choi, Pil Ja Seo, Byeng Wha Son, Uk Lee

**Affiliations:** a Department of Chemistry, Dongeui University, San 24 Kaya-dong Busanjin-gu, Busan 614-714, Republic of Korea; b Department of Chemistry, Pukyong National University, 599-1 Daeyeon 3-dong, Nam-gu, Busan 608-737, Republic of Korea

## Abstract

In the title compound, C_15_H_10_FIO_2_S, the O atom and the methyl group of the methyl­sulfinyl substituent are located on opposite sides of the plane through the benzofuran fragment. The 4-fluoro­phenyl ring is rotated out of the benzofuran plane by a dihedral angle of 28.33 (5)°. The crystal structure is stabilized by a weak non-classical inter­molecular C—H⋯O hydrogen bond and an I⋯O halogen interaction [3.211 (1) Å].

## Related literature

For the crystal structures of similar 2-(4-fluoro­phen­yl)-3-methyl­sulfinyl-1-benzofuran derivatives, see: Choi *et al.* (2009**a* ▶,b* ▶). For natural products with benzofuran ring systems, see: Akgul & Anil (2003 ▶); Soekamto *et al.* (2003 ▶). For the biological activity of benzofuran compounds, see: Aslam *et al.* (2006 ▶); Galal *et al.* (2009 ▶). For a review of halogen bonding, see: Politzer *et al.* (2007 ▶).
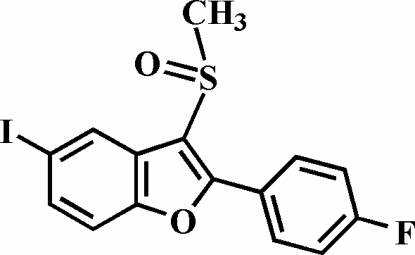


## Experimental

### 

#### Crystal data

C_15_H_10_FIO_2_S
*M* *_r_* = 400.19
Triclinic, 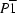

*a* = 8.1045 (2) Å
*b* = 8.2699 (2) Å
*c* = 11.0999 (3) Å
α = 94.538 (1)°
β = 91.118 (1)°
γ = 111.982 (1)°
*V* = 686.73 (3) Å^3^
*Z* = 2
Mo *K*α radiation
μ = 2.49 mm^−1^
*T* = 173 K
0.32 × 0.31 × 0.30 mm

#### Data collection

Bruker SMART APEXII CCD diffractometer
Absorption correction: multi-scan (*SADABS*; Bruker, 2009 ▶) *T*_min_ = 0.504, *T*_max_ = 0.526
12180 measured reflections
3179 independent reflections
3130 reflections with *I* > 2σ(*I*)
*R*_int_ = 0.024

#### Refinement

*R*[*F* ^2^ > 2σ(*F* ^2^)] = 0.018
*wR*(*F* ^2^) = 0.047
*S* = 1.17
3179 reflections
182 parameters
H-atom parameters constrained
Δρ_max_ = 0.32 e Å^−3^
Δρ_min_ = −0.90 e Å^−3^

### 

Data collection: *APEX2* (Bruker, 2009 ▶); cell refinement: *SAINT* (Bruker, 2009 ▶); data reduction: *SAINT*; program(s) used to solve structure: *SHELXS97* (Sheldrick, 2008 ▶); program(s) used to refine structure: *SHELXL97* (Sheldrick, 2008 ▶); molecular graphics: *ORTEP-3* (Farrugia, 1997 ▶) and *DIAMOND* (Brandenburg, 1998 ▶); software used to prepare material for publication: *SHELXL97*.

## Supplementary Material

Crystal structure: contains datablocks I. DOI: 10.1107/S1600536809051642/zq2022sup1.cif

Structure factors: contains datablocks I. DOI: 10.1107/S1600536809051642/zq2022Isup2.hkl

Additional supplementary materials:  crystallographic information; 3D view; checkCIF report

## Figures and Tables

**Table 1:** Hydrogen-bond geometry (Å, °)

*D*—H⋯*A*	*D*—H	H⋯*A*	*D*⋯*A*	*D*—H⋯*A*
C10—H10⋯O2^i^	0.95	2.53	3.432 (2)	158

Symmetry code: (i) 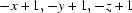.

**Fig. 1.**
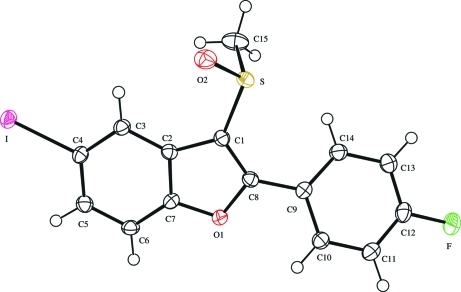
The molecular structure of the title compound with the atom numbering scheme. Displacement ellipsoids are drawn at the 50% probability level. H atoms are presented as a small spheres of arbitrary radius.

**Fig. 2.**
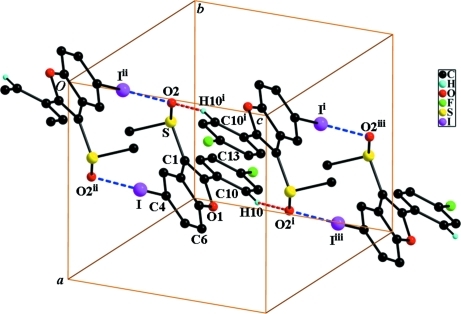
C—H···O and C—I···O interactions (dotted lines) in the crystal structure of the title compound. [Symmetry codes: (i) - x + 1, - y + 1, - z + 1; (ii) - x + 1, - y + 1, - z; (iii) x, y, z + 1.]

## supplementary crystallographic information

### Comment

Molecules involving benzofuran moiety have attracted widespread interest owing to their presence in natural products (Akgul & Anil, 2003; Soekamto *et al.*, 2003) and their biological activity (Aslam *et al.*, 2006; Galal *et al.*, 2009). As a part of our continuing studies of the effect of side chain substituents on the solid state structures of 2-(4-fluorophenyl)-3-methylsulfinyl-1-benzofuran analogues (Choi *et al.*, 2009*a,b*), we report the crystal structure of the title compound (Fig. 1).

The benzofuran unit is essentially planar, with a mean deviation of 0.010 (1) Å from the least-squares plane defined by the nine constituent atoms. The dihedral angle formed by the plane of the benzofuran and the 4-fluorophenyl ring is 28.33 (5)°. The crystal packing (Fig. 2) is stabilized by a weak non-classical intermolecular C—H···O hydrogen bond between the 4-fluorophenyl H atom and the oxygen of the S═O unit, with a C10—H10···O2^i^ (Table 1), and an I···O halogen bond between the iodine and the oxygen of the S═O unit [I···O2^ii^ = 3.211 (1) Å; C—I···O^ii^ = 170.98 (5)°] (Politzer *et al.*, 2007).

### Experimental

77% 3-Chloroperoxybenzoic acid (247 mg, 1.1 mmol) was added in small portions to a stirred solution of 2-(4-fluorophenyl)-5-iodo-3-methylsulfanyl-1-benzofuran (384 mg, 1.0 mmol) in dichloromethane (20 mL) at 273 K. After being stirred at room temperature for 3h, the mixture was washed with saturated sodium bicarbonate solution and the organic layer was separated, dried over magnesium sulfate, filtered and concentrated in vacuum. The residue was purified by column chromatography (hexane–ethyl acetate, 1:1 v/v) to afford the title compound as a colorless solid [yield 87%, m.p. 485–486 K; *R*_f_ = 0.66 (hexane-ethyl acetate, 1:1 v/v)]. Single crystals suitable for X-ray diffraction were prepared by slow evaporation of a solution of the title compound in chloroforrm at room temperature.

### Refinement

All H atoms were positioned geometrically and refined using a riding model, with C—H = 0.95 Å for aromatic H atoms and 0.98 Å for methyl H atoms, and with *U*_iso_(H) = 1.2*U*_eq_(C) for aromatic H atoms and 1.5*U*_eq_(C) for methyl H atoms.

### Crystal data

**Table:**

C_15_H_10_FIO_2_S	*Z* = 2
*M**_r_* = 400.19	*F*(000) = 388
Triclinic, *P*1	*D*_x_ = 1.935 Mg m^−^^3^
Hall symbol: -P 1	Mo *K*α radiation, λ = 0.71073 Å
*a* = 8.1045 (2) Å	Cell parameters from 9982 reflections
*b* = 8.2699 (2) Å	θ = 2.7–27.7°
*c* = 11.0999 (3) Å	µ = 2.49 mm^−^^1^
α = 94.538 (1)°	*T* = 173 K
β = 91.118 (1)°	Block, colourless
γ = 111.982 (1)°	0.32 × 0.31 × 0.30 mm
*V* = 686.73 (3) Å^3^	

### Data collection

**Table:**

Bruker SMART APEXII CCD diffractometer	3179 independent reflections
Radiation source: Rotating Anode	3130 reflections with *I* > 2σ(*I*)
HELIOS	*R*_int_ = 0.024
Detector resolution: 10.0 pixels mm^-1^	θ_max_ = 27.7°, θ_min_ = 1.8°
φ and ω scans	*h* = −10→10
Absorption correction: multi-scan (*SADABS*; Bruker, 2009)	*k* = −10→10
*T*_min_ = 0.504, *T*_max_ = 0.526	*l* = −13→14
12180 measured reflections	

### Refinement

**Table:**

Refinement on *F*^2^	Primary atom site location: structure-invariant direct methods
Least-squares matrix: full	Secondary atom site location: difference Fourier map
*R*[*F*^2^ > 2σ(*F*^2^)] = 0.018	Hydrogen site location: difference Fourier map
*wR*(*F*^2^) = 0.047	H-atom parameters constrained
*S* = 1.17	*w* = 1/[σ^2^(*F*_o_^2^) + (0.0241*P*)^2^ + 0.2941*P*] where *P* = (*F*_o_^2^ + 2*F*_c_^2^)/3
3179 reflections	(Δ/σ)_max_ < 0.001
182 parameters	Δρ_max_ = 0.32 e Å^−^^3^
0 restraints	Δρ_min_ = −0.89 e Å^−^^3^

### Special details

**Table:**

Geometry. All esds (except the esd in the dihedral angle between two l.s. planes) are estimated using the full covariance matrix. The cell esds are taken into account individually in the estimation of esds in distances, angles and torsion angles; correlations between esds in cell parameters are only used when they are defined by crystal symmetry. An approximate (isotropic) treatment of cell esds is used for estimating esds involving l.s. planes.
Refinement. Refinement of F^2^ against ALL reflections. The weighted R-factor wR and goodness of fit S are based on F^2^, conventional R-factors R are based on F, with F set to zero for negative F^2^. The threshold expression of F^2^ > 2sigma(F^2^) is used only for calculating R-factors(gt) etc. and is not relevant to the choice of reflections for refinement. R-factors based on F^2^ are statistically about twice as large as those based on F, and R- factors based on ALL data will be even larger.

### Fractional atomic coordinates and isotropic or equivalent isotropic displacement parameters (Å^2^)

**Table:**

	*x*	*y*	*z*	*U*_iso_*/*U*_eq_	
I	0.388646 (14)	0.197645 (13)	−0.026745 (9)	0.02319 (5)	
S	0.80967 (5)	0.78626 (5)	0.39558 (4)	0.01979 (9)	
F	1.07549 (17)	0.74121 (17)	0.98010 (11)	0.0369 (3)	
O1	0.65247 (16)	0.31158 (15)	0.51009 (11)	0.0203 (2)	
O2	0.67539 (17)	0.81288 (17)	0.31415 (13)	0.0269 (3)	
C1	0.7317 (2)	0.5608 (2)	0.41682 (15)	0.0185 (3)	
C2	0.6277 (2)	0.4171 (2)	0.33021 (15)	0.0176 (3)	
C3	0.5720 (2)	0.3991 (2)	0.20821 (15)	0.0195 (3)	
H3	0.6019	0.4976	0.1624	0.023*	
C4	0.4713 (2)	0.2317 (2)	0.15682 (15)	0.0195 (3)	
C5	0.4243 (2)	0.0842 (2)	0.22213 (16)	0.0218 (3)	
H5	0.3537	−0.0280	0.1834	0.026*	
C6	0.4802 (2)	0.1007 (2)	0.34311 (16)	0.0217 (3)	
H6	0.4509	0.0024	0.3889	0.026*	
C7	0.5807 (2)	0.2682 (2)	0.39309 (15)	0.0189 (3)	
C8	0.7428 (2)	0.4910 (2)	0.52316 (15)	0.0185 (3)	
C9	0.8296 (2)	0.5608 (2)	0.64234 (16)	0.0189 (3)	
C10	0.7577 (2)	0.4728 (2)	0.74357 (16)	0.0210 (3)	
H10	0.6515	0.3708	0.7340	0.025*	
C11	0.8411 (2)	0.5344 (2)	0.85731 (17)	0.0240 (3)	
H11	0.7935	0.4752	0.9262	0.029*	
C12	0.9944 (2)	0.6832 (2)	0.86850 (17)	0.0247 (4)	
C13	1.0692 (2)	0.7737 (2)	0.77148 (18)	0.0258 (4)	
H13	1.1747	0.8763	0.7823	0.031*	
C14	0.9860 (2)	0.7105 (2)	0.65755 (17)	0.0230 (3)	
H14	1.0360	0.7697	0.5891	0.028*	
C15	0.9920 (3)	0.7971 (3)	0.3035 (2)	0.0335 (4)	
H15A	0.9474	0.7159	0.2304	0.050*	
H15B	1.0779	0.7646	0.3491	0.050*	
H15C	1.0506	0.9166	0.2806	0.050*	

### Atomic displacement parameters (Å^2^)

**Table:**

	*U*^11^	*U*^22^	*U*^33^	*U*^12^	*U*^13^	*U*^23^
I	0.03081 (8)	0.02065 (7)	0.01672 (7)	0.00884 (5)	−0.00240 (5)	−0.00103 (4)
S	0.02125 (19)	0.01521 (18)	0.0224 (2)	0.00624 (15)	0.00127 (15)	0.00201 (15)
F	0.0418 (7)	0.0385 (7)	0.0225 (6)	0.0071 (5)	−0.0113 (5)	0.0010 (5)
O1	0.0245 (6)	0.0177 (6)	0.0171 (6)	0.0059 (5)	0.0003 (5)	0.0025 (4)
O2	0.0258 (6)	0.0245 (6)	0.0324 (7)	0.0108 (5)	−0.0005 (5)	0.0085 (5)
C1	0.0200 (7)	0.0166 (7)	0.0185 (8)	0.0067 (6)	0.0011 (6)	0.0009 (6)
C2	0.0188 (7)	0.0157 (7)	0.0188 (8)	0.0070 (6)	0.0021 (6)	0.0012 (6)
C3	0.0235 (8)	0.0182 (7)	0.0175 (8)	0.0086 (6)	0.0009 (6)	0.0026 (6)
C4	0.0225 (8)	0.0210 (8)	0.0159 (8)	0.0095 (6)	0.0008 (6)	0.0001 (6)
C5	0.0238 (8)	0.0190 (8)	0.0210 (9)	0.0067 (6)	0.0021 (6)	−0.0005 (6)
C6	0.0256 (8)	0.0175 (8)	0.0214 (9)	0.0067 (6)	0.0039 (6)	0.0049 (6)
C7	0.0212 (7)	0.0208 (8)	0.0154 (8)	0.0087 (6)	0.0022 (6)	0.0023 (6)
C8	0.0187 (7)	0.0168 (7)	0.0201 (8)	0.0066 (6)	0.0021 (6)	0.0015 (6)
C9	0.0200 (7)	0.0210 (8)	0.0177 (8)	0.0102 (6)	0.0014 (6)	0.0017 (6)
C10	0.0217 (8)	0.0217 (8)	0.0203 (8)	0.0091 (6)	0.0017 (6)	0.0020 (6)
C11	0.0280 (9)	0.0263 (9)	0.0196 (9)	0.0118 (7)	0.0022 (7)	0.0045 (7)
C12	0.0276 (9)	0.0273 (9)	0.0200 (9)	0.0122 (7)	−0.0057 (7)	−0.0004 (7)
C13	0.0220 (8)	0.0261 (9)	0.0257 (9)	0.0058 (7)	−0.0025 (7)	0.0006 (7)
C14	0.0212 (8)	0.0247 (8)	0.0220 (9)	0.0068 (7)	0.0014 (6)	0.0047 (7)
C15	0.0265 (9)	0.0312 (10)	0.0470 (13)	0.0128 (8)	0.0145 (9)	0.0157 (9)

### Geometric parameters (Å, °)

**Table:**

I—C4	2.096 (2)	C6—C7	1.380 (2)
I—O2^i^	3.211 (1)	C6—H6	0.9500
S—O2	1.491 (1)	C8—C9	1.455 (2)
S—C1	1.768 (2)	C9—C14	1.397 (2)
S—C15	1.792 (2)	C9—C10	1.403 (2)
F—C12	1.354 (2)	C10—C11	1.385 (3)
O1—C7	1.376 (2)	C10—H10	0.9500
O1—C8	1.381 (2)	C11—C12	1.378 (3)
C1—C8	1.370 (2)	C11—H11	0.9500
C1—C2	1.444 (2)	C12—C13	1.379 (3)
C2—C7	1.394 (2)	C13—C14	1.388 (3)
C2—C3	1.398 (2)	C13—H13	0.9500
C3—C4	1.385 (2)	C14—H14	0.9500
C3—H3	0.9500	C15—H15A	0.9800
C4—C5	1.401 (2)	C15—H15B	0.9800
C5—C6	1.388 (3)	C15—H15C	0.9800
C5—H5	0.9500		
			
C4—I—O2^i^	170.98 (5)	C1—C8—C9	135.10 (16)
O2—S—C1	107.38 (8)	O1—C8—C9	114.45 (14)
O2—S—C15	105.67 (9)	C14—C9—C10	119.35 (16)
C1—S—C15	98.36 (9)	C14—C9—C8	121.35 (16)
C7—O1—C8	106.78 (13)	C10—C9—C8	119.27 (15)
C8—C1—C2	107.00 (14)	C11—C10—C9	120.17 (17)
C8—C1—S	125.99 (13)	C11—C10—H10	119.9
C2—C1—S	126.68 (13)	C9—C10—H10	119.9
C7—C2—C3	119.08 (15)	C12—C11—C10	118.64 (17)
C7—C2—C1	105.32 (14)	C12—C11—H11	120.7
C3—C2—C1	135.60 (15)	C10—C11—H11	120.7
C4—C3—C2	117.23 (15)	F—C12—C11	118.06 (17)
C4—C3—H3	121.4	F—C12—C13	118.91 (17)
C2—C3—H3	121.4	C11—C12—C13	123.02 (17)
C3—C4—C5	122.69 (16)	C12—C13—C14	118.09 (17)
C3—C4—I	118.60 (12)	C12—C13—H13	121.0
C5—C4—I	118.71 (13)	C14—C13—H13	121.0
C6—C5—C4	120.44 (16)	C13—C14—C9	120.72 (17)
C6—C5—H5	119.8	C13—C14—H14	119.6
C4—C5—H5	119.8	C9—C14—H14	119.6
C7—C6—C5	116.26 (16)	S—C15—H15A	109.5
C7—C6—H6	121.9	S—C15—H15B	109.5
C5—C6—H6	121.9	H15A—C15—H15B	109.5
O1—C7—C6	125.22 (15)	S—C15—H15C	109.5
O1—C7—C2	110.46 (14)	H15A—C15—H15C	109.5
C6—C7—C2	124.30 (16)	H15B—C15—H15C	109.5
C1—C8—O1	110.43 (14)		
			
O2—S—C1—C8	−140.15 (15)	C1—C2—C7—C6	−179.85 (16)
C15—S—C1—C8	110.45 (17)	C2—C1—C8—O1	0.04 (18)
O2—S—C1—C2	32.41 (17)	S—C1—C8—O1	173.80 (12)
C15—S—C1—C2	−76.99 (16)	C2—C1—C8—C9	178.16 (17)
C8—C1—C2—C7	0.84 (18)	S—C1—C8—C9	−8.1 (3)
S—C1—C2—C7	−172.87 (13)	C7—O1—C8—C1	−0.91 (18)
C8—C1—C2—C3	−178.44 (18)	C7—O1—C8—C9	−179.46 (13)
S—C1—C2—C3	7.8 (3)	C1—C8—C9—C14	−28.7 (3)
C7—C2—C3—C4	0.3 (2)	O1—C8—C9—C14	149.37 (16)
C1—C2—C3—C4	179.47 (17)	C1—C8—C9—C10	153.29 (19)
C2—C3—C4—C5	0.3 (2)	O1—C8—C9—C10	−28.6 (2)
C2—C3—C4—I	−178.89 (11)	C14—C9—C10—C11	0.1 (2)
C3—C4—C5—C6	−0.8 (3)	C8—C9—C10—C11	178.20 (15)
I—C4—C5—C6	178.41 (13)	C9—C10—C11—C12	0.3 (3)
C4—C5—C6—C7	0.6 (2)	C10—C11—C12—F	−179.46 (16)
C8—O1—C7—C6	179.87 (16)	C10—C11—C12—C13	−0.2 (3)
C8—O1—C7—C2	1.47 (18)	F—C12—C13—C14	178.88 (16)
C5—C6—C7—O1	−178.21 (15)	C11—C12—C13—C14	−0.4 (3)
C5—C6—C7—C2	0.0 (3)	C12—C13—C14—C9	0.8 (3)
C3—C2—C7—O1	177.99 (14)	C10—C9—C14—C13	−0.7 (3)
C1—C2—C7—O1	−1.44 (18)	C8—C9—C14—C13	−178.75 (16)
C3—C2—C7—C6	−0.4 (3)		

Symmetry codes: (i) −*x*+1, −*y*+1, −*z*.

### Hydrogen-bond geometry (Å, °)

**Table:**

*D*—H···*A*	*D*—H	H···*A*	*D*···*A*	*D*—H···*A*
C10—H10···O2^ii^	0.95	2.53	3.432 (2)	158

Symmetry codes: (ii) −*x*+1, −*y*+1, −*z*+1.
